# ‘It’s not me, it’s them’ – a report describing the weight-related attitudes towards obesity in pregnancy among maternal healthcare providers

**DOI:** 10.1186/s12884-024-06591-z

**Published:** 2024-06-03

**Authors:** Taniya S. Nagpal, Kirina Angrish, Emily Bonisteel, Rebecca M. Puhl, Zachary M. Ferraro, Niyati M. Malkani, Caroline LeJour, Kristi B. Adamo

**Affiliations:** 1 Faculty of Kinesiology, Sport, and Recreation, University of Alberta, 116 St & 85 Ave, Edmonton, AB T6G 2R3 Canada; 2 Faculty of Applied Health Sciences, Brock University, St. Catharines, Canada; 3 Department of Human Development & Family Sciences, University of Connecticut, Storrs, CT USA; 4 Faculty of Medicine, University of Toronto, Toronto, Canada; 5 Department of Obstetrics and Gynecology, College of Medicine, University of Saskatchewan, Saskatoon, Canada; 6 Faculty of Medicine, University of Calgary, Calgary, Canada; 7 Faculty of Health Sciences, University of Ottawa, Ottawa, Canada

**Keywords:** Pregnancy, Obesity, Maternal health, Weight stigma, Attitudes, Prenatal care

## Abstract

**Background:**

Occurrences of weight stigma have been documented in prenatal clinical settings from the perspective of pregnant patients, however little is known from the viewpoint of healthcare providers themselves. Reported experiences of weight stigma caused by maternal healthcare providers may be due to negative attitudes towards obesity in pregnancy and a lack of obesity specific education. The objective of this study was to assess weight-related attitudes and assumptions towards obesity in pregnancy among maternal healthcare providers in order to inform future interventions to mitigate weight stigma in prenatal clinical settings.

**Methods:**

A cross-sectional survey was administered online for maternal healthcare providers in Canada that assessed weight-related attitudes and assumptions towards lifestyle behaviours in pregnancy for patients who have obesity. Participants indicated their level of agreement on a 5-point likert scale, and mean scores were calculated with higher scores indicating poorer attitudes. Participants reported whether they had observed weight stigma occur in clinical settings. Finally, participants were asked whether or not they had received obesity-specific training, and attitude scores were compared between the two groups.

**Results:**

Seventy-two maternal healthcare providers (midwives, OBGYNs, residents, perinatal nurses, and family physicians) completed the survey, and 79.2% indicated that they had observed pregnant patients with obesity experience weight stigma in a clinical setting. Those who had obesity training perceived that their peers had poorer attitudes (3.7 ± 0.9) than those without training (3.1 ± 0.7; t(70) = 2.23, *p* = 0.029, Cohen’s d = 0.86).

**Conclusions:**

Weight stigma occurs in prenatal clinical environments, and this was confirmed by maternal healthcare providers themselves. These findings support advocacy efforts to integrate weight stigma related content and mitigation strategies in medical education for health professionals, including maternal healthcare providers. Future work should include prospective examination of weight related attitudes among maternal healthcare providers and implications of obesity specific education, including strategies on mitigating weight stigma in the delivery of prenatal care.

## Background

Prenatal healthcare environments have been described as a prominent source of weight stigma experiences for pregnant patients with obesity [1–3]. Weight stigma, defined as societal devaluation of people with higher body weight and commonly expressed as negative weight-based stereotypes [4], can have a detrimental impact on biopsychosocial health outcomes, patient-provider rapport, and equitable access to healthcare [4–7]. The problem of weight stigma in clinical healthcare settings has been documented for two decades [8]. For example, providers attributing patients’ health issues to excess weight, expressing patronizing and disrespectful comments about patients’ weight and body size, and making assumptions about patients’ lifestyle behaviours (e.g., assuming physical inactivity, poor dietary choices, and treatment noncompliance) [9]. Emerging evidence demonstrates similar examples of weight stigma in prenatal contexts [2]. For instance, it is commonly noted that if a patient has obesity, prenatal healthcare providers more frequently ascribe potential perinatal complications to weight or obesity alone rather than investigating the etiology of the problem or further inquiring about the patient’s perspective on their concerns [2, 10]. Another example includes patients feeling uncomfortable discussing breastfeeding with healthcare providers because they may assume that the patients are unable to breastfeed due to having obesity, and so support will be inadequate [1]. Additionally, pregnant individuals with obesity have described interactions with providers where risks related to prenatal obesity have been communicated with blaming style language, leading patients to experience excessive worry and fear for fetal well-being even when no complications may be present [1, 2, 10].

The common social narrative surrounding prenatal obesity and excessive gestational weight gain circles back to assuming that obesity is solely caused by the pregnant person eating poorly and being inactive [11]. This narrow view ignores the multifactorial nature of positive energy balance and obesity predisposition [12]. Unfortunately, when providers reduce causes of obesity to intrapersonal factors alone, they are less likely to offer patient-centred care [13]. For example, a study with 248 maternity care providers reported that most of the participants felt pregnant populations who have obesity lack self-management behaviours [14]. Most maternal healthcare providers indicate that they feel unprepared to have sensitive weight-related discussions and desire more training on offering effective gestational weight management strategies [15, 16]. As maternal healthcare providers have several contact points with pregnant patients throughout the gestational period, it is necessary to ensure that their delivery of care is sensitive and free from stigmatization. Improving provider attitudes and preventing assumptions associated with obesity may be an important step towards mitigating weight stigma in prenatal clinical settings.

Previous studies have documented negative attitudes towards patients with obesity among a range of different health professionals including family physicians, physiotherapists, nurses, dietitians, and trainees (e.g., residents, physiotherapy students and nursing students) [17–25]. Strategies to mitigate weight stigma in clinical settings have centred around improving weight-related attitudes, and incorporating weight stigma and bias training as core competencies in medical curricula [26]. Education on weight stigma and bias includes understanding patient experience and learning how to engage in reflective practice to recognize one’s own biases and actively prevent them from interfering with delivering quality healthcare [26, 27]. In fact, healthcare providers who have received obesity-specific training, such as understanding the complex etiology of obesity beyond intrapersonal factors, recognition of weight stigma, and awareness of delivering sensitive care, have positive attitudes towards obesity and are more likely to be equipped to offer inclusive and patient-centred care [17, 24].

The primary objective of this exploratory study was to assess weight-related attitudes and assumptions towards obesity specifically among maternal healthcare providers. We also examined whether having had pregnancy-specific obesity education was related to better weight-related attitudes and less assumptions about maternal obesity compared to having no specialized training in this area. We hypothesized that negative attitudes towards obesity and poor assumptions about lifestyle behaviours in pregnancy would be present among maternal healthcare providers. We also predicted that healthcare professionals with pregnancy and obesity-specific training would have more positive attitudes than those without any specialized training in this area. These findings may support further advocacy of implementing effective strategies, such as education for maternal healthcare providers, to mitigate weight stigma experienced in prenatal clinical settings.

## Methods

This is a descriptive study. A cross-sectional survey was administered online using Qualtrics from January 2022 to May 2022. The Checklist for Reporting Results of Internet E-Surveys was followed to guide the methods and reporting of results [28]. The survey was promoted through social media platforms including Facebook and Twitter, with the advertisement indicating eligibility criteria and the purpose of the study to explore weight-related attitudes. Participants were eligible if they met the following criteria: (1) identify as a maternal healthcare provider (i.e., OBGYN, midwives, prenatal nurse, family physicians, or resident in training to be a maternal healthcare provider); (2) currently practicing in or completing their medical residency (if applicable) in Canada and; (3) able to read, write and understand English. Participants who met the eligibility criteria were directed to a letter of information, and after their review, they provided informed consent and completed the survey. An incentive to complete the survey included entry into a draw to win one of two $100 gift cards to Amazon Canada. This study was approved by the Brock University Research Ethics Board (21–117).

### Demographics

Participants reported their age, sex, province or territory they currently practice or are completing their residency in, and profession or profession they are pursuing if they are currently a resident. If participants had completed their residency or if residency was not applicable to their profession, they were asked to provide the number of years they had been practicing.

### Attitudes towards obesity in pregnancy

We modified the ‘Attitudes towards caring for patients who have obesity in pregnancy (Cronbach’s alpha α = 0.78)’, ‘Assumptions towards compliance with weight management (Cronbach’s alpha α = 0.65), and ‘Attitudes of colleagues and peers towards caring for pregnant patients who have obesity’ (Cronbach’s alpha α = 0.86) sub-scales developed by Puhl et al. (2014) [19] to be specific to pregnancy and gestational weight management. Participants indicated their level of agreement with each statement on a 5-point likert scale (1 = strongly disagree and 5 = strongly agree). Mean scores were calculated with a higher score indicating more negative attitudes. In addition, we asked participants to indicate whether they have ever known a pregnant person who has obesity and has experienced bias or discrimination in a healthcare setting because of their weight; response options were ‘yes’, ‘no’ or ‘prefer not to answer’ which were presented as a percentage.

### Assumptions about lifestyle behaviours for pregnant patients who have obesity

A 10-item questionnaire developed for the purpose of this study asked participants to rank, on a 5-point likert scale (1 = strongly disagree and 5 = strongly agree), their assumptions towards lifestyle behaviours in pregnancy, obesity and gestational weight management. The three lifestyle behaviours specified were sleep, physical activity, and nutrition, as they are most often stereotyped as individual causal factors of obesity and excessive gestational weight gain (11). Statements compared individuals with obesity with those who do not have obesity for each of these behaviours, and participants indicated their level of agreement with each statement. The mean score was calculated, and a higher score indicated worse assumptions towards lifestyle behaviours for patients who are pregnant and have obesity. Cronbach’s alpha for this questionnaire was α = 0.70.

### Obesity and pregnancy specific training

Participants were asked to indicate if they had received any specialized training to care for pregnant patients who have obesity (yes or no). If yes, an open-ended response option was available to describe their training. In addition, we applied a content analysis to the open-ended responses to code the data as specific or non-specific training. Two independent reviewers assessed the open-ended responses independently to decide if the training was specific or non-specific. Defined *a priori*, the response was coded as ‘specific’ if the training could be attributed directly to pregnancy and obesity (e.g., gestational weight counselling for patients who have obesity, procedural training for patients who have obesity such as care after bariatric surgery, delivery and labour care). Non-specific training was if it was unclear if the training was for managing care or understanding obesity in pregnancy, or was vaguely described as training (e.g., generally saying ‘modules’ or ‘attending a seminar’). If there were any discrepancies between the coding, a third reviewer was consulted for a final decision.

### Analysis

Demographic characteristics were summarized as means with standard deviation, and as percentages to describe the sample. Positively framed items were reverse-coded. Means and standard deviations were calculated for each scale, as well as percent agreement with each statement by combining responses of ‘agree’ and ‘strongly agree’ to further describe the population’s assumptions and attitudes. Student’s T-Test was used to compare scores based on whether participants had indicated receiving training for obesity in pregnancy. A sub-analysis was done to compare those with specific versus non-specific training. Significance was accepted as *p* < 0.05. Effect sizes were calculated referring to Cohen’s (1988) criteria: Cohen’s d values for t-tests (small: 0.2, medium: 0.5, large: 0.8). All analyses were performed with SPSS Version 27.

## Results

A total of 94 participants indicated eligibility and completed the consent form, of which 75 progressed to survey items. Three individuals did not complete any survey items and were therefore excluded from the analysis (*N* = 72).

The average respondent age was 39.0 (± 11.6) years. Most of the respondents identified as female (83%) and almost half were practicing or training in Ontario (44%). Most had completed their training (74%), while 19% were in their residency. Among those practicing, they had been in their profession for an average of 9.5 years (± 13.6). Across professions 37 were OBGYNs (51.4%), 19 were family physicians (26.4%), 10 participants were midwives (13.9%), 4 were prenatal nurses (5.6%), and 2 participants selected ‘prefer not to answer’ (2.8%). Thirty-three participants indicated they had obesity training (45.8%), of which 18 were coded as specific training for obesity in pregnancy (78.0%) and 15 (22.0%) were non-specific. All demographic characteristics are summarized in Table 1.

**Table 1:** Description of study population

Characteristic	*N* = 72
Age (average years ± SD)	39.0 ± 11.6
Sex (*n*; %)	
Female	60 (83.3)
Male	11 (15.3)
Non-Binary	0 (0.0)
Prefer Not to Answer	1 (1.4)
Profession/Prospective Profession if Resident (*n*; %)	
OBGYN	37 (51.4)
Midwife	10 (13.9)
Family Physician	19 (26.4)
Prenatal Nurse	4 (5.9)
Prefer Not to Answer	2 (2.8)
Residents in Training (*n*; %)	14 (19.4)
OBGYN	13 (92.8)
Family Physician	1 (7.2)
Years Practicing Profession (average years ± SD)	9.5 ± 13.6
Location Presently Practicing or In Residency (*n*; %)	
British Columbia	7 (9.7)
Alberta	24 (33.3)
Saskatchewan	3 (4.2)
Manitoba	2 (2.8)
Ontario	32 (44.4)
Quebec	1(1.4)
New Brunswick	1 (1.4)
Nova Scotia	1 (1.4)
Newfoundland	1(1.4)
Specialized Training in Pregnancy and Obesity (*n*; %)	
Yes	33 (45.8)
Specific	18 (54.5)
Non-Specific	15 (45.5)
No	39 (54.2)

Note: Participants who indicated they are presently completing their residency are not included in the average calculation for ‘years practicing profession’. Specificity of training assessed via content analysis. SD-Standard deviation

Average scores on each of the scales are presented in Table 2. Participants who had received obesity training scored higher (3.7 ± 0.9) on the scale evaluating attitudes of colleagues and peers towards caring for pregnant patients who have obesity (t(70) = 2.23, *p* = 0.029, Cohen’s d = 0.86), than those who did not have training (3.1 ± 0.7). This suggests that those who have training evaluated their peers and colleagues as having worse attitudes towards caring for pregnant patients who have obesity than those who did not have training. No other significant differences emerged. Table 3 presents the scores based on whether participants indicated they had received specialized training, as well as whether that training was specific or non-specific.

**Table 2:** Average score on scales

Scale	Average Score
Assumptions about lifestyle behaviours for pregnant patients who have obesity	2.1; 0.7
Attitudes towards caring for patients who have obesity in pregnancy	2.3; 0.5
Assumptions towards compliance with gestational weight management	2.4; 0.5
Attitudes of colleagues and peers towards caring for pregnant patients who have obesity	3.4; 0.9

Note. All scores are presented as means; standard deviation. All scales were assessed on a 5-point likert scale where 1 means strongly disagree and 5 means strongly agree, a higher score would be indicative of a negative attitude or poor assumptions

**Table 3:** Scores based on having received obesity and pregnancy specific training

Scale	With Training *N* = 33	Without Training *N* = 39	Effect Size
Assumptions about lifestyle behaviours for pregnant patients who have obesity	2.0; 0.6	2.1; 0.7	0.68
Attitudes towards caring for patients who have obesity in pregnancy	2.4; 0.6	2.1; 0.5	0.56
Assumptions towards compliance with gestational weight management	2.3; 0.6	2.4; 0.5	0.51
Attitudes of colleagues and peers towards caring for pregnant patients who have obesity*	3.7; 0.9	3.1; 0.7	0.86

**Scale**	**Specific Training** ***N*** **= 18**	**Non-Specific Training** ***N*** **= 15**	**Effect Size**
Assumptions about lifestyle behaviours for pregnant patients who have obesity	2.0; 0.7	2.2; 0.5	0.62
Attitudes towards caring for patients who have obesity in pregnancy	2.3; 0.6	2.5; 0.6	0.58
Assumptions towards compliance with gestational weight management	2.2; 0.6	2.4; 0.4	0.56
Attitudes of colleagues and peers towards caring for pregnant patients who have obesity	3.7; 1.0	3.5; 0.9	1.0

Note. All scores are presented as means; standard deviation. All scales were assessed on a 5-point likert scale where 1 means strongly disagree and 5 means strongly agree, a higher score would be indicative of a negative attitude or poor assumptions. *p < 0.05

When asked whether they have known a pregnant person who has obesity and has experienced bias or discrimination in healthcare, 57 (79.2%) indicated yes, 12 (16.7%) said no, and 3 selected prefer not to answer (4.1%). Table 4 presents each scale and percent agreement with each item. A few key descriptions include while 79% of participants believed they provide quality care to their patients who have obesity, only 11% felt that they are professionally prepared to do so suggesting a gap in the training they receive. Regarding lifestyle behaviours, 22% of participants agreed that their pregnant patients who have obesity are not likely to engage in exercise or healthy nutrition habits, and 12% indicated they dislike caring for obesity in pregnancy. Furthermore, most participants (70%) stated they have heard or witnessed a colleague make a negative remark about a pregnant patient who has obesity, and half of the sample agreed that other healthcare professionals are not equipped for caring for obesity in pregnancy.

**Table 4:** Individual scales with percent agreement with each item

Scale and Items	% Agreement *N* = 72
*Assumptions about lifestyle behaviours for pregnant patients who have obesity*	
My patients who are pregnant and have obesity likely do not exercise compared to my patients without obesity.	22.2
My patients who are pregnant and have obesity are not likely to be motivated to improve their exercise habits compared to patients without obesity.	11.1
My patients who are pregnant and have obesity likely do not have good sleep habits (e.g., set bedtime and wake up time) compared to patients without obesity.	8.3
My patients who are pregnant and have obesity are not likely to be motivated to improve their sleep habits compared to patients without obesity.	5.5
My patients who are pregnant and have obesity likely do not meet nutritional recommendations compared to patients without obesity.	23.6
My patients who are pregnant and have obesity are not likely to be motivated to improve their nutrition habits compared to patients without obesity.	11.1
I am just as comfortable discussing gestational weight gain with a patient who has a normal weight body mass index compared to obesity.	59.7
I am just as comfortable discussing exercise recommendations with a patient who has a normal weight body mass index compared to obesity.	72.2
I am just as comfortable discussing sleep recommendations during pregnancy with a patient who has a normal weight body mass index compared to obesity.	76.4
I am just as comfortable discussing nutrition recommendations during pregnancy with a patient who has a normal weight body mass index compared to obesity.	81.9
*Attitudes towards caring for patients who have obesity in pregnancy*	
I often feel frustrated with patients who have obesity during their pregnancy.	15.2
Patients who are pregnant and have obesity can be difficult to deal with.	25.0
I feel that it is important to treat patients who are pregnant and have obesity with compassion and respect.	100.0
I dislike treating patients who are pregnant and have obesity	12.5
I see no difference between a patient who is pregnant and has an obese or normal weight body mass index.	45.8
I feel confident that I provide quality care to my patients who are pregnant and have obesity.	79.2
I feel professionally prepared to effectively care for my patients who are pregnant and have obesity	11.1
I feel that patients who are pregnant and have obesity are often non-compliant with my recommendations.	8.3
I feel that patients who are pregnant with obesity lack motivation to make lifestyle changes.	11.1
Caring for patients who are pregnant and have obesity is professionally rewarding.	12.5
Patients who are pregnant and have obesity tend to be lazy.	6.9
*Assumptions towards compliance with gestational weight management*	
Patients who are pregnant and have obesity are receptive to gestational weight management advice.	61.1
Patients who are pregnant and have obesity are compliant with gestational weight management advice.	33.3
Patients who are pregnant and have obesity are motivated to change their diet.	40.2
Patients who are pregnant and have obesity can be successful in making dietary changes during their pregnancy.	80.5
I have confidence that patients who are pregnant and have obesity can stay within gestational weight gain recommendations.	48.6
I enjoy counseling and working with patients who are pregnant and have obesity.	58.3
*Attitudes of colleagues and peers towards caring for pregnant patients who have obesity*	
Other health providers in my field often have negative stereotypes toward patients with obesity during pregnancy.	70.8
I have heard/witnessed other professionals in my field make negative comments about patients with obesity during pregnancy.	70.8
My colleagues tend to have negative attitudes toward patients with obesity during pregnancy.	38.8
Health care providers feel uncomfortable when caring for patients who are pregnant and have obesity	52.7

Note. Percentages include participants who selected ‘agree’ or ‘strongly agree’ with the statement

## Discussion

This descriptive study assessed attitudes and assumptions about obesity in pregnancy among maternal healthcare providers. Most of the participants indicated that they have known a pregnant person with obesity who has experienced weight stigma in a healthcare setting. Although most participants expressed confidence in their ability to provide quality care to their pregnant patients who have obesity, more than 50% of the respondents suggested that their peers and colleagues may be subjecting patients to weight stigma. Most participants also highlighted not feeling professionally prepared to manage obesity in pregnancy, suggesting a critical gap in training. Moreover, those who received obesity training reported poorer attitudes of their peers and colleagues towards caring for prenatal obesity than those who did not have training, suggesting the value of obesity-specific training in recognizing potential weight-stigmatizing behaviours. Previous studies have documented patient perspectives of weight stigma in prenatal clinical settings [1–3, 16], and our findings reiterate the presence of weight stigma in prenatal care now also from the perspective of maternal healthcare providers themselves.

To reduce weight stigma in prenatal clinical settings, opportunities for adequate training and education on recognizing weight stigma, sensitive discussions about gestational weight gain, and reflective practices to identify one’s own biases are needed. Some studies have demonstrated improved attitudes among non-prenatal healthcare professionals following active self-reflection as well as educational interventions aimed at increasing awareness of weight biases [29, 30]. Maternal healthcare providers should be offered insight and feedback on potentially stigmatizing practices they may be engaging in, whether implicitly or explicitly, which may be negatively perceived by patients. In pregnancy, commonly cited sources of weight stigma include the way a provider discusses gestational weight gain, such as suggesting that patients are engaging in health behaviours associated with weight gain like physical inactivity and poor diet [10]. In our participant pool, it appeared that most healthcare providers do not assume a pregnant patient who has obesity has poorer health behaviours (i.e., physical activity, nutrition or sleep) than those who do not have obesity. However, only 40% of them agreed that their patients who have obesity will meet gestational weight gain recommendations and are motivated to make diet related changes. To improve attitudes and reduce assumptions about health behaviours, training for maternal healthcare providers should include an understanding of obesity’s complex etiology that extends beyond individual control and empathetic conversational skills for discussing gestational weight gain [13, 16].

Providers should be offered skills-based training to discuss gestational weight gain from a patient-centred level [2]. Using tools like the 5 As for discussing gestational weight gain may help providers offer patient-centred care [31]. This framework empowers patients to lead the discussion surrounding gestational weight gain and positions the professional as an advocate for their goals and needs [31]. When providers discuss gestational weight gain free from biases using frameworks like the 5 As, patients are more likely to be receptive, actively engage with recommendations, build trust with their provider and therefore have a better overall clinical experience and potentially health outcome [31]. Moving forward, future work may include developing modules on weight stigma and bias specific to maternity care and testing their effectiveness in improving attitudes among maternal healthcare providers.

Strengths of this study include the diverse inclusion of maternal healthcare professionals, and high compliance to completing all survey items. The sub-scale assessing attitudes towards compliance, however, had low internal consistency and this should be interpreted with caution. Limitations include the use of convenience sampling and resultant majority of respondents identifying as female and residing in Ontario and Alberta. As well, we used social media for recruitment and therefore a response rate cannot be calculated. Additionally, selection bias is possible given those with an interest in obesity or weight stigma may have been more likely to complete the survey. There may have been reporting bias as the subject of weight stigma is sensitive and providers may not be trained in effective self-reflection or, despite anonymity, provided a socially desirable response to indicate positive attitudes. To prevent selection and reporting biases, future work may include integrating assessments and training opportunities within curricula or continuing education to reach a wider audience. Further research is needed to understand maternal healthcare provider attitudes towards obesity in pregnancy with a larger and more diverse sample. Overall, these findings provide initial evidence from the perspective of maternal healthcare providers in Canada that weight stigma continues to exist in prenatal clinical settings, and strategies to prevent this from occurring need to be tested and implemented.

## Conclusions

Maternal healthcare providers expressed generally positive attitudes towards obesity in pregnancy and limited negative assumptions about lifestyle behaviours like physical activity, nutrition, and sleep. However, most respondents reported that they have observed weight stigma occur within the healthcare setting and that their peers and colleagues have poor attitudes towards pregnant patients who have obesity. Future work is needed to design and test prenatal care specific training modules on weight stigma and bias and assess the effect on improving provider attitudes.

## Data Availability

No datasets were generated or analysed during the current study.
